# Hand is a direct target of the forkhead transcription factor Biniou during *Drosophila *visceral mesoderm differentiation

**DOI:** 10.1186/1471-213X-7-49

**Published:** 2007-05-18

**Authors:** Dmitry Popichenko, Julia Sellin, Marek Bartkuhn, Achim Paululat

**Affiliations:** 1 Department of Biology, University of Osnabrück, Zoology, Barbarastraße 11, D-49069 Osnabrück, Germany; 2 Institute for Genetics, Justus-Liebig-Universität Gießen, D-35390 Gießen, Germany

## Abstract

**Background:**

The visceral trunk mesoderm in *Drosophila melanogaster *develops under inductive signals from the ectoderm. This leads to the activation of the key regulators Tinman, Bagpipe and Biniou that are crucial for specification of the circular visceral muscles. How further differentiation is regulated is widely unknown, therefore it seems to be essential to identify downstream target genes of the early key regulators. In our report we focus on the analysis of the transcriptional control of the highly conserved transcription factor Hand in circular visceral muscle cells, providing evidence that the *hand *gene is a direct target of Biniou.

**Results:**

Herein we describe the identification of a regulatory region in the *hand *gene essential and sufficient for the expression in the visceral mesoderm during embryogenesis. We found that *hand *expression in the circular visceral mesoderm is abolished in embryos mutant for the FoxF domain containing transcription factor Biniou. Furthermore we demonstrate that Biniou regulates *hand *expression by direct binding to a 300 bp sequence element, located within the 3^rd ^intron of the *hand *gene. This regulatory element is highly conserved in different *Drosophila *species. In addition, we provide evidence that Hand is dispensable for the initial differentiation of the embryonic visceral mesoderm.

**Conclusion:**

In the present report we show that cross species sequence comparison of non-coding sequences between orthologous genes is a powerful tool to identify conserved regulatory elements. Combining functional dissection experiments *in vivo *and protein/DNA binding studies we identified *hand *as a direct target of Biniou in the circular visceral muscles.

## Background

In *Drosophila*, the visceral midgut musculature consists of two layers of myofibers that derive from different embryonic primordia. The inner layer of circular muscles originates from a subset of cells of the so-called trunk mesoderm and is characterized, e.g., by the expression of the bHLH factor Hand [1-5]. The outer lattice of longitudinal muscles arises from caudal mesoderm, located at the posterior tip of the blastoderm anlagen and is characterized by the expression of, e.g., bHLH54F [6]. Two cell types contribute to the formation of the circular muscles: founder cells (fc) and fusion competent myoblasts (fcm). During development, the founder cells fuse with the neighboring fusion competent myoblasts to form binucleated myofibers that elongate to surround the endodermal midgut later on [7-9]. Recently it was shown that fusion in the visceral mesoderm depends on receptor tyrosine kinase signaling [10-13], whereas further differentiation depends on molecules including, e.g., Blown fuse and Kette [14].

The visceral trunk mesoderm, as part of the early dorsal mesoderm, develops under inductive signals mediated by Decapentaplegic (Dpp) [15]. Dpp is essential but not sufficient for the selection and differentiation of progenitors that give rise to cardioblasts, pericardial cells, several dorsal somatic muscles and the midgut circular muscles. Additional mesoderm-intrinsic factors are indispensable to enable cells to respond to the external signal. A key player in the differentiating dorsal mesoderm cells is the NK homeobox transcription factor Tinman (Tin), which is activated as a response to Dpp signaling. Loss of Tinman activity results in the absence of all derivatives of the dorsal mesoderm, including heart and circular visceral muscles [16,17]. Further development of the visceral trunk mesoderm requires the activity of the downstream factors Bagpipe (Bap, NK homeobox transcription factor) and Biniou (Bin, FoxF forkhead domain transcription factor), which are initially coexpressed in specific patches of cells in a segmental pattern along the anteroposterior axis of the dorsal mesoderm [15,16,18,19]. Tinman and Bagpipe appear transiently in the visceral mesoderm and their activity diminishes during further visceral differentiation, indicating that both genes are responsible primarily for visceral mesoderm specification rather than differentiation. Biniou was shown to be crucial for further differentiation rather than cell specification. Biniou mutant embryos display visceral mesodermal cells but fail to form differentiated midgut musculature [19,20]. The activity of several genes depends on Biniou, including *fasciclin III*, *brokenheart*, *vimar*, *dpp *and *β3Tubulin *[19,21]. Regulation of *dpp *and *β3tubulin *in the visceral trunk mesoderm requires direct binding of Biniou to specific enhancer elements, whereas the other downstream genes might be regulated indirectly.

In this report we examined the regulation of the bHLH transcription factor Hand in the circular visceral mesoderm. Hand is expressed at stage 11 in the specified circular visceral muscle progenitors [3], thus after the initial activity of the key regulators Bap and Bin. Using functional dissection assays *in vivo*, combined with a sequence comparison approach among *hand *loci of closely related *Drosophila *species as well as protein/DNA binding studies, we identified a highly conserved 300 bp element (Hand Visceral, HV-element), located in the 3^rd ^intron of the *hand *gene, which is crucial for activation of *hand *in circular visceral muscles. Our biochemical studies showed that the FoxF-transcription factor Biniou binds directly to the HV-element. Together with the observation that *hand *expression is abolished in the visceral mesoderm of *bin *mutant embryos whereas being normal in other expression domains, e.g., in the heart, our results indicate that *hand *is a direct target of Biniou in the visceral trunk mesoderm. Embryos homozygous mutant for the *hand *gene show no morphological abnormalities in visceral mesoderm development, indicating that Hand function is dispensable for the initial steps of visceral differentiation.

## Results and discussion

### Visceral expression of *hand *depends on a 300 bp intron sequence

**Figure 1:**
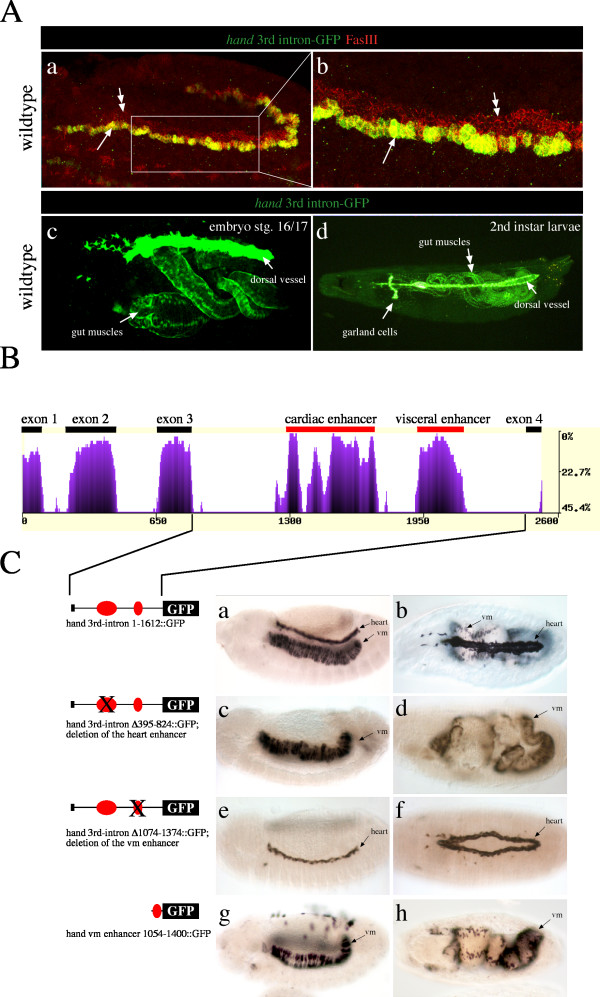
**Identification of regulatory elements responsible for *hand *expression in the visceral mesoderm**. A) *hand-GFP expression in the wildtype*. The third intron of the *hand *gene harbors all cis-regulatory sequences sufficient to control GFP reporter gene expression in circular visceral muscles. (a) Double labeling was performed using an anti-GFP (green channel) and an anti-FasciclinIII (red channel) antibody. Fasciclin III stains the progenitors as well as the fusion competent myoblasts (double arrows) that form the circular muscles. Solely the progenitor cells coexpress *hand *driven GFP at the stage shown here (arrows). (b) is an enlargement of a (boxed). (c) *hand *driven GFP expression is maintained through embryonic and larval development. The stage 16/17 embryo shown (lateral view) reveals *hand *driven GFP expression in the heart and the circular visceral midgut muscles. (d) shows a 2^nd ^larvae exhibiting direct GFP fluorescence in the nuclei of the circular visceral muscles, the heart and the garland cells. The diffuse yellow color inside the gut is caused by autofluorescence from yeast. B) *eShadow analysis for the hand gene*. The alignment of multiple sequences was performed with the ClustalW algorithm implemented in the eShadow web application [41]. Sequences from *D. melanogaster*, *D. erecta*, *D. yakuba*, *D. simulans *and *D. virilis *were used. Exons and conserved intron sequences, identified to harbor enhancer elements driving *hand *in the heart and the visceral mesoderm, are marked. The x-axis corresponds to size in base pairs. The y-axis corresponds to percentage of variation in a window of 50 bp. C) Highly conserved regions in the 3^rd ^intron of the *hand *gene are responsible for driving expression in cardiac tissue and within the visceral mesoderm. All embryos were stained with an anti-GFP antibody. a, c, e and g show embryos at stage 13, lateral view. b, d, f and h show stage 16/17 embryos in a dorsal view. (a and b) Transgenic embryos carrying the whole 3^rd ^intron of the *hand *gene reveal expression of the reporter gene in the developing heart and circular visceral mesoderm. Garland cells, which express *hand*-GFP as well, are out of focus. (c and d) Transgenic embryos with a mutated 3^rd ^intron, lacking the first highly conserved element (region 395–824), show a total loss of reporter gene expression in the heart, whereas expression in the visceral mesoderm is unaffected. (e and f) Transgenic embryos with a mutated 3^rd ^intron, lacking the second highly conserved element (region 1074–1374) show a total loss of reporter gene expression in the visceral mesoderm, whereas expression in the cardiac mesoderm is unaffected. (vm) circular visceral muscles. (g and h) Transgenes carrying the visceral mesoderm enhancer (region 1054–1400) fused to GFP show expression in the visceral mesoderm but not in garland cells, lymph glands and the heart.

Within the visceral trunk mesoderm, Hand is first detectable after the activation of the early key regulators Bagpipe (Bap), Tinman (Tin) and Biniou (Bin) [19], raising the possibility that *hand *might be regulated by one of these factors. To investigate this hypothesis we have searched for regulatory regions that are crucial for driving *hand *expression in the primordium of the circular visceral muscles. The 3^rd ^intron of the *hand *gene harbors all regulatory enhancers sufficient to drive *hand *expression in cardiac cells during embryogenesis and all postembryonic stages, as shown previously [1,5]. In addition, the 3^rd ^intron is capable to activate reporter gene expression in the visceral mesoderm (see Figure 1A). Reporter gene expression starts in stage 11 embryos in the progenitors of the circular visceral muscles, but is excluded from the visceral fusion competent cells [3]. With beginning fusion, *hand*-GFP expression is detectable in syncytial visceral myofibers and persists until end of embryogenesis. In addition, visceral expression of *hand*, as mimicked by the reporter gene activity, is present in larvae, pupae and adult flies (Figure 1A and unpublished data).

To identify the enhancer elements crucial for the expression of *hand *in the visceral mesoderm, we compared *hand *3^rd ^intron sequences of orthologous loci in closely related *Drosophila *species, an approach which was shown previously to be highly efficient to identify conserved regulatory elements [22,23]. Therefore we amplified the 3^rd ^intron from *D. erecta*, *D. mauritiana*, *D. simulans*, *D. teissieri*, and *D. yakuba*, which was possible because the 3^rd ^intron is flanked by highly conserved exons, allowing us the use of slightly degenerated primers (see Methods section for details). In addition, the complete sequence of the *hand *locus from several Drosophilids became available as part of recent genome sequencing projects. Sequence comparison of the *hand *3^rd ^intron from various species revealed two highly conserved sequence blocks (Figure 1B). To test whether one or both of these conserved elements have functional relevance we compared the capability of full-length 3^rd ^intron and of mutated 3^rd ^intron sequences to drive reporter gene expression in transgenic *D. melanogaster *flies. The first construct harbors a deletion of 429 bp (region 395–824), thus removing the first highly conserved sequence block. Transgenic flies carrying the deletion construct exhibit a total loss of embryonic reporter gene expression in cardiomyoblasts, pericardial and lymph gland cells but not in visceral trunk mesoderm (Figure 1C), indicating that the first conserved sequence block is required for *hand *expression in heart cells. These findings are consistent with the observation of Han and coworkers, who recently identified a cardiac and hematopoietic enhancer (HCH enhancer) in the same gene region [1]. Han and coworkers showed that activation of the *hand *gene in heart and hematopoietic cells is regulated, at least partially, through the activity of the transcription factors Tinman, Pannier and Serpent that bind directly to the HCH enhancer.

The second construct we tested carries a deletion of 300 bp corresponding to the second large conserved sequence block in the *hand *3^rd ^intron (region 1074–1374). Transgenic flies carrying this construct exhibit a total loss of reporter gene expression in the visceral trunk mesoderm, whereas cardiac expression remains unaffected (Figure 1C). Furthermore, fusion of the visceral enhancer element to a reporter gene shows that it is capable to drive reporter gene activity in a pattern identical to that of endogenous *hand *expression in the visceral mesoderm. Thus, the HV-enhancer is not only necessary but also sufficient to drive reporter gene activity in the visceral trunk mesoderm and, later on in development, in the circular visceral muscles (Figure 1C). We conclude that the regulation of *hand *in the visceral mesoderm is accomplished by regulatory elements located in a conserved 300 bp sequence element (named Hand Visceral, HV-element).

### Biniou activates *hand *in the visceral mesoderm by direct binding to the HV regulatory region

**Figure 2:**
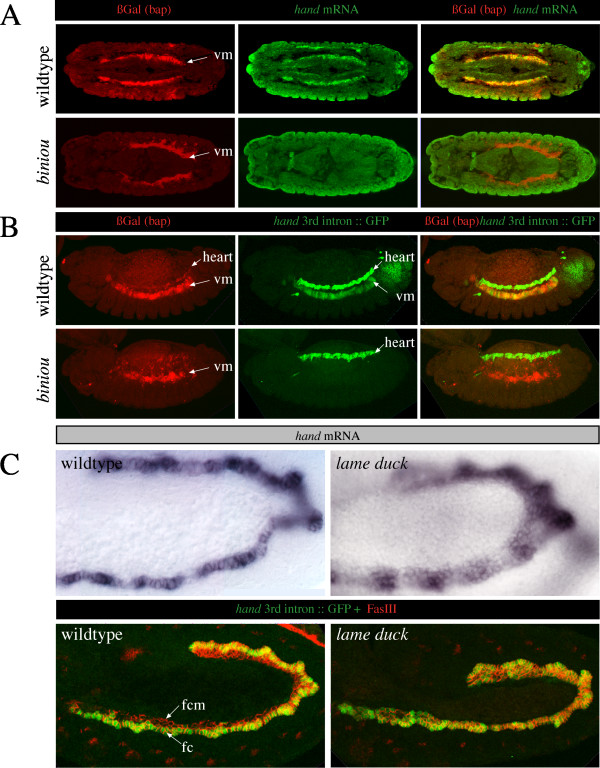
**Biniou is a regulator of *hand *in the visceral mesoderm**. A) Biniou is a regulator of *hand *expression *in vivo*. Shown are wildtype embryos carrying a *bap*-lacZ transgene (upper row), stained with a βGal antibody (in red) to visualize visceral mesodermal cells, and *hand *RNA (in green). Embryos lacking functional Biniou reveal a total loss of *hand *expression in the visceral mesoderm (lower row). (B) shows the result of a similar experiment. Here, the *hand *3^rd ^intron, fused to GFP, was used as a reporter. Embryos lacking Biniou reveal a complete absence of *hand*-GFP reporter gene expression in the visceral mesoderm. (C) Compared to the wildtype, embryos homozygous mutant for the Gli-like transcription factor Lame duck exhibit an expansion of *hand *expression in the visceral mesoderm (fc = founder cells, fcm = fusion competent myoblasts, vm = visceral mesoderm).

**Figure 3:**
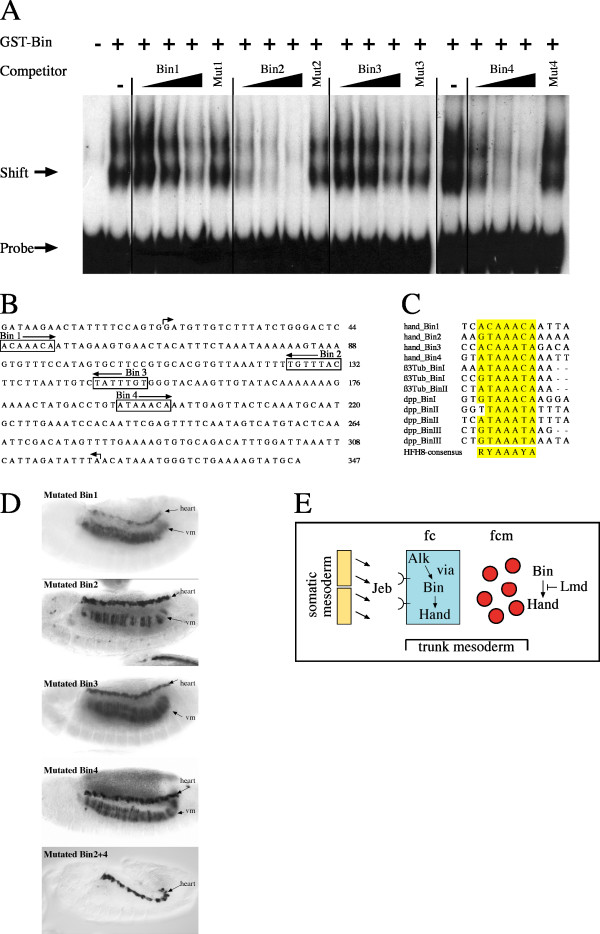
**Identification of Biniou binding sites in the *hand *visceral enhancer**. (A) Gel mobility shift assays with the HV-element as probe. Competition experiments using a 20- 200- and 2000-fold excess of unlabeled wildtype and a 2000-fold excess of mutated oligonucleotides, corresponding to the Biniou binding sites Bin1, Bin2, Bin3 and Bin4, were performed to reveal specificity. (B) Shown is the *D. melanogaster *visceral mesoderm enhancer region. Boxes label the four Biniou core binding site motifs, respectively. Arrows indicate the beginning and end of the deletion in the tested construct *hand*-3^rd^-intronΔ1074–1374::GFP (see result section). Same region was used for EMSA. (C) Sequence alignment of known Biniou binding sites, including the HFH-8 core consensus binding site. The colored nucleotides match the HFH-8 consensus sequence. β3Tub Biniou binding sites are from [21], Dpp Biniou binding sites are from [19]. (vm) visceral mesoderm; (lg) lymph glands. (D) Transgenic embryos carrying the whole 3^rd ^intron of the *hand *gene but with mutated Biniou binding sites as indicated (see methods section for details). Only the combination of mutated Bin2 and Bin4 sites lead to a total loss of reporter gene expression in the visceral mesoderm while expression in the heart is unaffected. (E) Model for the regulation of *hand *in visceral mesoderm. The activation of *hand *in the visceral mesoderm depends on Jeb/Alk signaling [10,11,29] and is likely mediated by the direct binding of Biniou to the identified visceral enhancer. Our results furthermore indicate that *hand *activity might be repressed in the visceral fusion compentent cells by the Gli-like transcription factor Lame duck (fc = founder cells, fcm = fusion competent myoblasts).

The experiments described above have confirmed that the highly conserved non-coding sequences located in the 3^rd ^intron of the *hand *gene are functional as cis-regulatory elements. Furthermore, the 3^rd ^intron of *hand *loci isolated from related Drosophilids are capable to drive reporter gene expression in transgenic *Drosophila melanogaster*, as shown by Han and coworkers for the HCH enhancer from *D. virilis *[1] and by us for the full-length intron isolated from *D. mauritiana *(own unpublished data). An additional observation indicates that the regulation of *hand *in cardiac and visceral mesoderm is conserved among Drosophilids. *D. erecta *and *D. yakuba *wildtype embryos, which we selected as examples, were probed with a labeled *hand *specific probe isolated from these species (for details see Methods section). In both species, *hand *expression is detectable in the visceral and cardiac mesoderm (data not shown). Our observations prompted us to search for upstream transcriptional activators responsible for visceral expression of *hand *by searching for conserved binding site motifs within the identified visceral enhancer. Alignment of sequences with homology to the *hand *visceral enhancer revealed the presence of four putative motifs with homology to the HFH-8 consensus binding site A/G C/T A A A C/T A [24], which is recognized by the forkhead domain transcription factor Biniou [19]. The location of the four potential Biniou binding sites is depicted in Figure 3B. To verify whether the expression of *hand *indeed depends on the activity of Biniou, we examined *hand *expression and *hand *driven GFP reporter gene expression in mutant embryos. In embryos lacking Biniou, *hand *expression is totally abolished in the visceral mesoderm (Figure 2A and 2B). The embryos we tested carried a *bap*-lacZ transgene, which serves as a marker for early visceral mesoderm [19,21]. Anti-βGalactosidase staining demonstrates that visceral mesodermal cells are present in *biniou *mutant embryos. Therefore, the absence of *hand *expression is not caused by the absence of these cells. To examine whether Biniou acts as a direct regulator on the HV-enhancer, we performed *in vitro *DNA-binding experiments (Figure 3A). Our results from gel mobility shift assays show that all four oligonucleotides (Bin1, Bin2, Bin3 and Bin4) corresponding to the four putative Biniou binding sites in the HV-element can compete for binding of Biniou, although with different specificity. Oligonucleotides, in which the Forkhead core-binding motif was mutated, failed to compete.

We next analyzed the functionality of the four Biniou binding sites *in vivo*. For this purpose we established constructs carrying the *hand *3^rd ^intron, but with individually mutated Biniou binding sites, fused to a reporter gene. We found that none of the tested mutations causes a total loss of reporter gene activity in the visceral mesoderm (Figure 3D). Nevertheless, a mutated Bin2 site results in a reduced reporter gene expression in the trunk mesoderm. Reporter gene expression in the cardiac mesoderm, serving as an internal control, appears normal. Next we tested a construct in which the two binding sites that reveal highest binding specificity in the gel mobility shift assay, Bin2 and Bin4, were simultaneously mutated. This results in the total loss of enhancer activity in the embryonic visceral mesoderm, while expression in other tissues remains unaffected (Figure 3D). Our observations indicate that the combined binding of the Bin2 and Bin4 sites is critical for transcriptional activation of *hand *in the embryonic visceral mesoderm.

The high conservation of the 300 bp visceral enhancer element among Drosophilids prompted us to search for additional regulators. Therefore we searched for transcription-factor binding sites by *in silico *analysis using various web-based algorithms (e.g., MatchTM) and a candidate approach. The only potentially interesting binding site, predicted by such algorithms, indicates binding of Hairy. Thus, we analyzed the expression of *hand *in the corresponding *hairy *mutant and found that *hand *expression is not altered. Because of their known expression in visceral cells, we considered other transcription factors as being potential regulators of *hand*, although binding sites were not predicted in the visceral enhancer. When analyzing embryos homozygous mutant for the Gli-like transcription factor Lame duck (Lmd), we observed a strong expansion of *hand *expression in the visceral mesoderm (Figure 2C). Lmd is described as one of the major factors crucial for myoblast fusion [25-28]. In the visceral mesoderm Lmd is expressed in the fusion competent cells. In contrast to its function in the somatic mesoderm as a positive regulator of fusion, essentially by promoting downstream factors like Sticks and Stones (Sns), it is dispensable for fusion in the visceral mesoderm [28]. Our results indicate that Lmd acts directly or indirectly as a repressor of *hand *at early stages within the fusion competent cells prior to fusion. At developmental stages when *hand *is normally restricted to a single row of visceral founder cells we found *hand *to be generally expressed in all cells of the visceral mesoderm in the *lmd *mutant (Figure 2C).

In a recent study, Varshney and Palmer provided evidence that tyrosine kinase anaplastic lymphoma kinase (Alk)-mediated signaling in the developing *Drosophila *gut positively drives *hand *transcription [29], although through an unknown effector. In Alk mutant embryos *hand *expression is abolished in the visceral mesoderm while being normal in other tissues, for instance the heart. Overexpression of Jelly belly (Jeb), the corresponding Alk ligand [10,11], within the entire mesoderm, induces Alk signaling in all cells of the visceral mesoderm, and therefore leads to an expanded expression of *hand *in these cells. The authors point out that several candidate molecules exist which might regulate the transcription of *hand *in the visceral mesoderm in response to Alk activation, among them is Biniou. Our present work clearly demonstrates that Biniou is indeed critical for *hand *regulation in the visceral mesoderm.

### Hand function during visceral mesoderm differentiation

**Figure 4:**
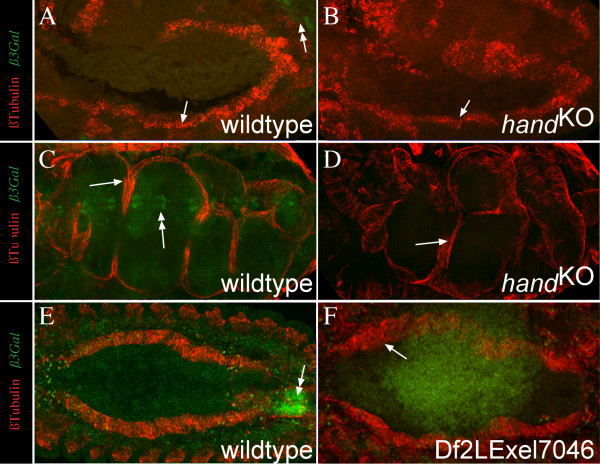
**Hand is dispensable for initial midgut differentiation**. (A-F) show embryos stained for β3Tubulin (red channel, mesodermal derivatives), and βGalactosidase (green channel, balancer staining, marked with double arrows). Embryos with mutated *hand *gene, either induced by homologues recombination (*hand*KO) or by a deficiency (DF(2L)^Exel7046^) reveal normal formation of visceral mesoderm and a differentiated gut at later stages (D). A, C and E show wildtype embryos for comparison. A and B show stage 10 embryos from a lateral view, C and D stage 16/17 embryos from a dorsolateral view and E and F stage 13/14 embryo from a dorsal view. The embryo shown in F reveals weak autofluorescence do to overexposure of the green channel to clearly demonstrate the absence of the balancer.

Since the absence of Biniou activity affects differentiation of the midgut visceral mesoderm, and expression of *hand *is lost in the trunk mesoderm as a consequence of the loss of Biniou function, we were interested to analyze whether Hand is required for midgut visceral muscle differentiation. We utilized the recently described *hand *specific mutant generated by homologous recombination [2] as well as the molecularly characterized deficiency Df(2L)^Exel7046 ^[30] that deletes 16 genes including *hand *(Flybase ID FBab0037918). When homozygous mutant embryos were examined for visceral mesoderm development, no detectable abnormalities were observed. Circular muscles, visualized by β3tubulin expression, are visible and the gut constrictions are formed normally as well (Figure 4). Our findings are consistent with the recent observations of Varshney & Palmer who used the deficiency Df(2L)^Exel7046 ^for a phenotypic analysis [29]. They show that neither Alk, nor Dumbfounded (Duf) or Sns, typical markers for visceral mesoderm, are misregulated in the absence of Hand. A lack of a visible morphological phenotype in the gut formation argues clearly against an early role of Hand in specification or initial differentiation of visceral mesoderm development, but leaves the possibility for a potential role of Hand as a transcriptional regulator in this tissue, presumably later throughout life cycle. This assumption is consistent with the high level of *hand *activity in postembryonic stages [5]. Examples of such physiological functions are mutations in the *vimar *and the *β3tubulin *gene [31,32]. Mutations in these genes cause lethality during larval stages without visible morphological abnormalities.

## Conclusion

To get further insights into the network of transcriptional control we have analyzed the activation of the bHLH transcription factor Hand. We found that the expression of *hand *in cells that give rise to the circular visceral muscles is directly controlled by the FoxF transcription factor Biniou. We show that two out of four Biniou binding sites, located within a 300 bp visceral enhancer element are crucial for hand regulation. Examination of *hand *mutant embryos revealed that *hand *is not required for initial steps of circular muscle formation during embryogenesis. Furthermore, the Gli-like transcription factor Lame Duck is needed to restrict *hand *expression to the founder cells of the visceral mesoderm at early stages. A model for the regulation of *hand *in the visceral mesoderm is provided in Figure 3E.

## Methods

### Fly stocks

Oregon R was the wild type used. Transgenic *Drosophila *lines were established by use of the w1118-line. *D. teissieri*, *D. yakuba*, *D. erecta *and *D. mauritiana *were kindly provided by Gunther Reuter (Halle, Germany) and *D. simulans *and *D. virilis *by Veiko Krauß (Leipzig, Germany). The null allele *bin*Il and *bap3*-lacZ were described in [19] and kindly provided by Manfred Frasch (Erlangen, Germany). The null allele *hand*-ko-2 was described in [2] and kindly provided by Zhe Han (Ann Arbor). The allele *lmd *was described in [25] and kindly provided by Hanh Nuygen (Erlangen). The deficiency Df(2L)^Exel7046 ^(BL 7819) was obtained from Bloomington Stock. Recently Han and coworkers showed that this deficiency deletes *hand *[2]. The used *hand *driven GFP lines were described previously [5,33] or described in the main text.

### Gene cloning

cDNAs from *D. erecta*, *D. mauritiana*, *D. simulans*, *D. teissieri*, *D. yakuba *were amplified with moderately degenerated primers (forward: 5'-tacagc(g/t)aa(c/t)aaaaa(a/g)ga-3'; reverse: gttc(a/c)gatgcccaaacatc-3') using the One StepRT-PCR-Kit (Qiagen, Hilden, Germany). Total RNA isolated from adult flies was used as template. The third intron from all species was amplified with the following primers (forward: 5'-attaaaacatt(a/g)aa(a/g)ttggc-3' and reverse: gttc(a/c)gatgcccaaacatc-3'). All PCR-amplicons were generally cloned into pCRII-Topo (Invitrogen) and sequenced for both strands.

### Embryo staining

In situ hybridization was carried out essentially as described [34], but with the use of digoxigenin-labeled RNA probes and an anti-Dig antibody (Roche Molecular Biochemicals). Riboprobes were generated using the *hand *cDNA from the different Drosophilid species. Collection of eggs and fixation was identical to the common methods used for *Drosophila melanogaster*. The following primary antibodies were used in this study: Guinea-pig anti-β3Tubulin (1:5000, gift from Renate Renkawitz-Pohl), mouse-anti FasciclinIII (7G10, 1:20, Developmental Studies Hybridoma Bank), mouse anti-βGalactosidase (1:2000; Promega-Z378A); rabbit anti-GFP (1:1000; Abcam-ab6556). All non-fluorescent antibody detections were performed using Vectastain Elite kit (Vector) and DAB staining. Confocal microscopy was performed using a Zeiss LSM Pascal microscope.

### Generation of GFP reporter gene constructs

Reporter constructs were made by ligating the regions of interest into pH-Stinger [35] and verified by partial sequencing. P-element-mediated germline transformations were performed as described [36]. Construct plasmid DNA was coinjected with Δ2–3 helper plasmid DNA [37] into *w*^- ^embryos. After backcrossing, three to five independent lines were examined for reporter expression per construct. The following *hand *reporter constructs were used in this study:

*hand *3^rd ^intron 1–1612::GFP (= *hand*-C-GFP), *hand *visceral enhancer 1054–1400::GFP, *hand *3^rd ^intron Δ395–824::GFP, *hand *3^rd ^intron Δ1074–1374::GFP, *hand *3^rd ^intron Bin1mut::GFP (the sequence TCACAAACAATT was replaced by TCACCTAGGATT), *hand *3^rd ^intron Bin2mut::GFP (the sequence TTTGTTTACTTTC was replaced by TTTACCGGTTTC), *hand *3^rd ^intron Bin3mut::GFP (the sequence TCTATTTGTGGG was replaced by TCTTCCGGAGGG), *hand *3^rd ^intron Bin4mut::GFP (Bin4mut = the sequence TGTATAAACAAAT was replaced by TGTACGTACGATT), *hand *3^rd ^intron Bin2mut4mut::GFP (Bin2mut = the sequence TTTTGTTTACTTC was replaced by TTTTACCGGTTTC, Bin4mut = the sequence TGTATAAACAAAT was replaced by TGTACGTACGATT).

### DNA binding assay

Protein synthesis, isolation and band shift assays were performed using standard protocols [38]. Purified Biniou GST fusion protein was used. The Bin/pGex3T plasmid was kindly provided by Manfred Frasch, New York. A 350 bp PCR-fragment, corresponding to the HV-enhancer shown in Figure 3B, was used as radiolabeled probe. For the gel mobility shift assays the HV-element was used as probe. Competition experiments were performed using a 20-, 200- and 2000-fold excess of unlabeled wildtype and a 2000-fold excess of mutated oligonucleotides. Oligos used for band shift experiments were:

Bin1 (5'-CTGGGACTCACAAACAATTAGAAGT-3'),

Mut1 (5'-CTGGGACTCATGGGACATTAGAAGT-3'),

Bin2 (5'-TAAATTTTTGTTTACTTCTTAATT-3'),

Mut2 (5'-TAAATTTTTTGGGCATTCTTAATT-3'),

Bin3 (5'-TTAATTGTCTATTTGTGGGTACAA-3'),

Mut3 (5'-TTAATTGTCGCGGGATGGGTACAA-3'),

Bin4 (5'-ATGACCTGTATAAACAAATTGAGT-3') and

Mut4 (5'-ATGACCTGTGCGGGTAAATTGAGT'-3').

### Sequence analysis

The high conservation of the coding region of *hand *allows the doubtless identification of the correct orthologues. Standard alignments were made with ClustalX and displayed using SeqVu. For cross-species comparison of the 3^rd ^intron sequences we used the following algorithms: Dialign [39], VISTA [40] and eShadow [41], all of which gave essentially the same results.

## Authors' contributions

DP identified the regulatory region responsible for expression of *hand *in the visceral mesoderm and has generated and tested the mutant *hand *reporter constructs. JS has performed the *in situ *hybridization of *hand *in *biniou *and *lmd *mutant background. MB carried out the EMSA. AP conceived and designed the study, and participated in the analysis of embryos at the confocal microscope and drafting the manuscript. All authors have read and approved the final manuscript.
